# Endometriosis Support and Development of Digital Technology–Based Interventions: Systematic Review

**DOI:** 10.2196/71859

**Published:** 2025-10-14

**Authors:** Tivizio Pavic, Kévin Nadarajah, Alain Somat, Geneviève Cabagno, Florence Terrade

**Affiliations:** 1 Laboratoire de Psychologie: Cognition, Comportement, Communication (LP3C) Univ Rennes Rennes France; 2 Équipe Recherche Psychologie Appliquée (PsyCAP), Centre d’Études et d’Expertise sur les Risques, l’Environnement, la Mobilité et l’Aménagement (Cerema) Saint-Brieuc France; 3 Valeurs, Innovations, Politiques, Socialisations et Sports (VIPS2) Univ Rennes Rennes France; 4 Centre d’études et de recherches en psychopathologie et psychologie de la santé (CERPPS) Toulouse France

**Keywords:** endometriosis, digital health interventions, technological intervention, endometriosis care, development framework, systematic review

## Abstract

**Background:**

Endometriosis is a chronic disease that affects 1 in 10 women worldwide. The disease affects patients’ daily life at physical, psychological, and social levels. In recent years, the management of this disease has evolved, thanks in particular to the emergence of digital technologies and associated interventions. However, despite their growing use, there seems to be no systematic review of their development, design, and efficacy.

**Objective:**

A systematic review was conducted with the aim of characterizing the development process, design, and effectiveness of interventions using a digital tool for endometriosis.

**Methods:**

A total of 7 databases (MEDLINE, APA PsycArticles, Academic Search Premier, Psychology and Behavioral Sciences Collection, APA PsycInfo, SocINDEX, and SPORTDiscus) were searched to identify relevant articles published between 2010 and 2024. The articles selected were analyzed using a methodological framework specific to the development of digital health interventions (Design and Evaluation of Digital Health Interventions [DEDHI]), consisting of 4 phases: preparation (phase 1, specific to application development), optimization (phase 2, dedicated to identifying the best intervention configurations), evaluation (phase 3, aiming to confirm the effectiveness of the intervention), and implementation (phase 4, implementing and updating the intervention on a large scale).

**Results:**

A selection of 10 articles was made from the 381 studies retrieved from the databases. Among these 10 studies, 6 distinct digital health interventions were identified. The interventions based on digital devices produced physical and psychological benefits. Analysis using the DEDHI framework showed (1) a disparity in the responses to the different phases (ie, 9/10, 90% of studies responding to phase 1; 3/10, 30% to phase 2; 4/10, 40% to phase 3; and 2/10, 20% to phase 4) and (2) a variability in the completion of the evaluation criteria ranging from 10% (1/10) to 80% (8/10) in phase 1, 0% (0/13) to 77% (10/13) in phase 2, 0% (0/10) to 80% (8/10) in phase 3, and finally 0% (0/13) to 77% (10/13) in phase 4. The objectives of these digital interventions were to support pain management (5/6, 83%), to provide information about the disease and strategies for managing it (4/6, 67%), and to provide psychosocial support (2/6, 33%).

**Conclusions:**

This systematic review highlights an emerging literature, limited regarding the use of digital technology in the management of endometriosis, and heterogeneous concerning the methodologies used. This variability limits the generalizability of the results and requires a nuanced interpretation of the available data. However, the results of this review have demonstrated the value of digital technology–based interventions to support endometriosis, while highlighting the importance of a methodological framework to structure their development to optimize patient support.

## Introduction

Endometriosis is a chronic disease characterized by the abnormal presence of an epithelium, stroma, or both, histologically similar to the endometrium, located outside the endometrium and myometrium, and frequently associated with an inflammatory state [1,2]. Its prevalence is estimated at around 10%; that is, approximately 190 million women of childbearing age worldwide [3]. Endometriosis is currently presented by major organizations such as the WHO, the European Parliament, the Office on Women’s Health, and the scientific community as a major public health problem [4]. Although responsible for significant morbidity, endometriosis is a disease for which there is as yet no effective treatment [5].

Lack of treatment has a deleterious impact on patients’ quality of life [6]. Thus, numerous physical constraints, such as chronic pelvic pain [7]; associated symptoms such as dysmenorrhea, dysuria, dyschezia, and dyspareunia [8]; and a greater predisposition to the risk of comorbidities [9] arise from this disease. As well as these physical impacts, endometriosis can also cause anxiety or depression [10], psychosomatic disorders [11], reduced self-esteem [12], or a negative perception of one’s body [13]. Finally, endometriosis can also affect these women’s social relationships, as it severely restricts their daily and professional lives [14], causes difficulties in their relationships and sexuality [15,16], and can, in some cases, lead to isolation [17]. The diversity of these symptoms and their varying degrees of severity in each woman can make universal management difficult. It is, therefore, important that the support offered to patients is based on a personalized approach (ie, a care pathway tailored to each patient), multidisciplinary (ie, close collaboration between different health care professionals), and multimodal (ie, several types and sources of support) [18].

Chronic disease support is embedded in a global context, where digital technology is playing an increasingly important role in health care [19]. Especially since the COVID-19 pandemic, digital technologies are receiving more and more attention as a relevant solution to improve chronic disease management [20,21]. Digital technologies can involve a wide range of aspects such as telemedicine, mobile health apps, internet, websites, virtual reality, artificial intelligence, distance education, etc [22-24]. The growth of these technologies offers new possibilities for digital interventions designed to support the development of knowledge, behavioral changes, etc [25]. For chronic disease, a dominant approach is to use these technologies to increase patients’ autonomy in managing their symptoms [26]. They can be used to improve patients’ quality of life [27,28] and allow them to be in charge of certain aspects of their health, such as symptom management [29].

Endometriosis has not escaped this digital wave, as several countries, including the Australian and French governments, have already introduced national plans based on these technologies to combat the disease. These plans aim to develop interventions to enhance the quality of life for women with endometriosis through the use of digital technology, both to raise awareness and to improve patient care. According to Giudice et al [30], there is an urgent need to invest in digital technologies (eg, mobile apps) dedicated to the management of endometriosis. Digital technologies can help to prevent the risks associated with the disease, involve different professionals in the care pathway, and adapt the care offered to the needs of each patient. Since 2013, enthusiasm for digital technologies addressing endometriosis has grown, with at least 26 health applications on platforms like the Apple iTunes Store or Google Play. These applications are designed to help patients manage their symptoms, provide information on diagnosis and treatment, act as a social network, and allow them to share their experiences or keep a health diary [31]. Although there is strong interest among researchers, politicians, and developers in interventions that use digital technologies to support chronic conditions, such as endometriosis [30,32,33], there is also evidence showing that these technologies do not necessarily deliver the expected positive health outcomes [34]. A lack of specific guidelines dedicated to the development and evaluation of technologies could explain this inefficiency [35,36]. Thus, methodological frameworks such as the iterative Design and Evaluation of Digital Health Interventions (DEDHI) [37] have been developed to support the long-term implementation of a digital technological innovation dedicated to health. Nevertheless, this type of framework still seems to have limited use [38,39], although it can be useful for guiding the development of innovations dedicated to newly studied pathologies, as is the case for endometriosis [4,37].

Scientists, politicians, and decision-makers are tending to promote the use of digital technologies, and applications dedicated to endometriosis are multiplying. However, to the authors’ knowledge, there is no systematic review on digital and technological interventions to support the management (ie, pain, mental health, knowledge, behavior, etc) of endometriosis. This systematic review aims to examine how digital health interventions (DHIs) targeting endometriosis have been developed and designed and to evaluate their effectiveness, with a particular focus on the use of a structured methodological framework and reported outcomes related to physical and psychological health.

## Methods

### Protocol

This systematic review followed the recommendations of the Preferred Reporting Items for Systematic Reviews and Meta-Analyses (PRISMA) checklist (Multimedia Appendix 1) [40,41]. The protocol of this review has been registered in the International Prospective Register of Systematic Reviews (PROSPERO CRD42023461263).

### Inclusion Criteria

The inclusion criteria for the studies are as follows: (1) participants must have endometriosis, (2) use digital technology solutions to manage endometriosis, (3) take place between 2010 and 2024, to reflect current technological development [42], and (4) be written in French or English.

### Search Strategy

The following computer databases were searched: Academic Search Premier, MEDLINE, APA PsycArticles, Psychology and Behavioral Sciences Collection, APA PsycInfo, SocINDEX, and SPORTDiscus. A total of 3 filters were used when searching the databases: years (2010-2024), peer-reviewed academic journals, and the terms appearing in the title or abstract. In order to make the research equation as exhaustive as possible, particularly with regard to digital tools, it was decided to draw on equations from recent research specifically on technological and digital interventions in the field of mental health [23]. These databases were examined using the following search equation: “endometriosis” AND (websites OR “smartphone app*” OR wearable OR “virtual reality” OR “augmented reality” OR “immersive technology” OR platform OR mhealth OR “mobile health” OR ehealth OR “e-mental health” OR e-health OR internet OR mail OR chat OR SMS OR text message OR digital intervention OR technological intervention) NOT (systematic review or meta analysis or scoping review or literature review or umbrella review). Databases were searched using the same equation through the Rennes 2 University library portal, which provides access to a wide range of bibliographic resources. In July 2024, the search equation was run for the first time and identified 5 articles in the final selection. In line with best practice for systematic reviews (eg, see chapter 4 of the Cochrane Handbook) [43], which recommends rerunning the search before final analysis to capture all newly published studies, the same search equation was rerun in November 2024 before submission. This update ensured the completeness and timeliness of the review before submission. The updated search identified a further 5 articles.

### Study Selection

As proposed by the PRISMA methodology [41], the screening stages were followed. Zotero software (Digital Scholar) [44] was used to extract references from databases. Rayyan software (developed by Ouzzani et al [45]) was used for the first selection steps, that is, duplicate identification and selection by title and abstract [46].

After removing duplicates, the titles and abstracts of all identified articles were examined for eligibility. Articles were excluded if (1) endometriosis was not mentioned, (2) participants were not ill, (3) it was a systematic review, exploratory review, or meta-analysis, (4) it did not mobilize digital technology or focus on an intervention mobilizing digital technology, (5) it was a research protocol, (6) it was written in a language other than French or English, and (7) it was written before 2010.

Potentially eligible articles were retrieved, and if the text was not available, the authors were contacted. All the texts retrieved were then examined by 2 reviewers (TP and KN). Any disagreements were resolved by discussion with a third author (GC).

### Risk of Bias

The risk of bias of each study included in this review was assessed with the Mixed Methods Assessment Tool (MMAT) [47]. A percentage was calculated for each study as proposed by Pluye et al [48] using the following formula: (number of “yes” responses divided by the number of “relevant criteria”) × 100. “Yes” indicates that an item is satisfied. A higher score indicates a lower risk of bias. The risk of bias was analyzed by 2 reviewers. A general intraclass correlation coefficient was calculated to determine interrater reliability. As recommended by Hong et al [47], no study, not even one of low methodological quality, was excluded. Interjudge agreement was satisfactory (0.98, 95% CI 0.82-0.99). Percentage scores are reported in Table 1.

**Table 1:** Study characteristics for included studies (n=10).

Specific digital tool	Authors	Country	Methods	Diagnosis sample size, n	Digital technologies	Risk of bias^a^, n (%)	Objective	
**Isolated initiative of an intervention test using a digital tool**								
	Abdulai et al [49]	Canada	Mixed methods	Patients diagnosed with or clinically suspected of having endometriosis (n=12)	Website	4.5 (90)	Evaluate the usability of the website and assess for destigmatizing properties of sexual health–related web-based resources.	
	Li et al [50]	Australia	Case study	N/A^b^	development of the information recommendation functionalities on the website	2 (40)	Presentation of a methodology for developing and implementing health information recommendation functions within web-based health applications, using the example of a website on endometriosis.	
	Lutfi et al [51]	Australia	Quantitative pilot RCTs^c^	Endometriosis diagnosis (n=19; telehealth=7, VR^d^=8, control=4)	Telehealth (1 h) vs VR (1 h) vs control (continue with their activities of daily living)	3.5 (70)	Determine the immediate impact of a single session of “supervised” telehealth-delivered exercise compared to “self-managed” VR-delivered exercise on pelvic pain associated with endometriosis.	
**Testing an intervention using virtual reality** **(Endocare)**								
	Merlot et al [52]	France	Quantitative RCTs	MRI^e^ endometriosis Diagnosis (n=45; Endocare=23, control=22)	VR vs control (same situation but in 2D on a tablet)	3.5 (70)	Measure the immediate and 4-hour persisting effects of a single use 20-minute DTx^f^ (Endocare) on pain in women experiencing pelvic pain due to endometriosis.	
	Merlot et al [53]	France	Quantitative RCTs	Endometriosis diagnosis (n=102; Endocare=51, control=51)	VR vs control (same situation but in 2D on a tablet)	3.5 (70)	Assess the effects of repeated at-home administrations of a 20-minute (VR) solution (Endocare) compared with a sham condition on pain in women experiencing pelvic pain due to endometriosis.	
**Testing an intervention using a mobile app (Endo-App)**								
	Rohloff et al [54]	Germany	Quantitative	Prior diagnosis of endometriosis (n=106; Endo-APP=64, no user=42)	App	3.5 (70)	Examine whether there is evidence of beneficial effects of the smartphone app “Endo-App” and whether a multicenter randomized controlled trial should be planned to substantiate these effects.	
	Rohloff et al [55]	Germany	Quantitative pilot RCTs	Medical diagnosis of endometriosis (n=122)	App + standard care vs usual care	4.5 (90)	Examine the impact of the Endo-App on both disease-related quality of life and symptoms of endometriosis affecting it.	
	Zugaj et al [56]	Germany	Qualitative	Patients diagnosed with endometriosis and who have received a prescription for the DiGa^g^ application: “Endo-App” (n=10)	App	5 (100)	Investigates how a health care app can influence the subjective experience of illness in patients with endometriosis.	
**Testing an intervention using SMS text messaging (EndoSMS)**								
	Sherman et al [57]	Australia	Mixed methods	Diagnosed with endometriosis (self-reported; n=17)	SMS text messaging	4 (80)	Co-design and evaluate the acceptability, readability, and quality of a bank of supportive SMS text messages (EndoSMS) for individuals with endometriosis.	
	Sherman et al [58]	Australia	Mixed methods integrated into RCTs	Clinically diagnosed with endometriosis (n=225; SMS=110, waitlist=115)	SMS text messaging	4.5 (90)	Determine the feasibility and acceptability of EndoSMS, a psychologically focused SMS text messaging intervention designed to support individuals living with endometriosis.	

^a^A higher percentage indicates a lower risk of bias.

^b^N/A: not available.

^c^RCT: randomized controlled trial.

^d^VR: virtual reality.

^e^MRI: magnetic resonance imaging.

^f^DTx: digital therapeutics.

^g^DiGa: digital health applications.

### Analysis of Articles Using the Framework for Designing and Evaluating DHIs

The DEDHI framework [37] was used to characterize the overall development process of digital technology–based interventions proposed in the studies of this corpus. Indeed, this framework seems particularly appropriate for the development of interventions based on digital technologies, specific guidelines dedicated to the design, and large-scale implementation of health-related innovations [59,60]. This framework proposes four stages: (1) the “preparation phase” describes the design phases of the innovation—review of the knowledge justifying an intervention, development of a conceptual model, and feasibility or acceptability study to test new components of the health intervention while identifying the optimization criterion offering the best result. This phase, therefore, requires a prototype offering the basic functionalities. (2) The “optimization phase” suggests building an optimized health care intervention using microtrials to identify the best configuration for the innovation, while respecting the optimization criteria. This phase requires a prototype offering all the functionalities set out in the conceptual model. (3) The “evaluation phase” is used to confirm the effectiveness of a health intervention, in particular, by carrying out a randomized controlled trial. At this stage, the prototype must be fully functional. And (4) the “implementation phase” offers a guide for the large-scale implementation and maintenance of an effective health intervention. For each phase, different criteria need to be addressed in order to optimize the long-term use of health innovations and recommended levels of technological maturity. They are organized according to 3 axes into each phase: goals and tasks (from 1 to 4 criteria, eg, feasibility and acceptability in the first phase), technical maturity (1 criterion, eg, elaborated research prototype in phase 2), and evaluation (from 5 to 10 criteria, eg, adherence, safety, and personalization). The 2 reviewers (TP and KN) independently examined the presence or absence of each criterion in the articles, and a third reviewer (GC) was called in in the event of disagreement. This made it possible to calculate a percentage of completion for each phase, determined by the ratio between the number of criteria present and the total number of criteria expected for that phase.

## Results

**Figure 1:**
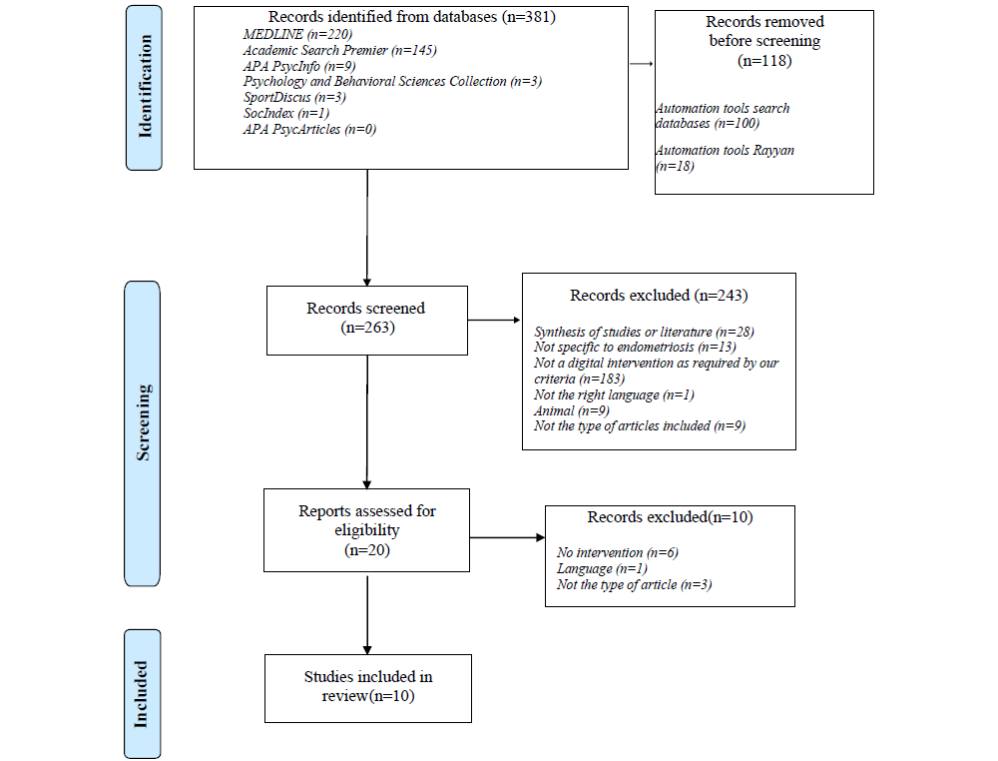
PRISMA (Preferred Reporting Items for Systematic Reviews and Meta-Analyses) flow diagram.

Figure 1 illustrates the screening stages in the PRISMA flow diagram.

### Studies Design

Of the 10 studies included, 3 (30%) used mixed methods [49,57], one of which was a pilot randomized controlled trial [58]. One study was based on a qualitative analysis [56], and 5 (50%) studies were solely based on quantitative analyses [51-55], 4 of which were randomized controlled trials [51-53,55]. Finally, a study on the development of a methodology for designing a digital tool was based on a case study of endometriosis [50]. For this reason, due to its general methodology, this article will not be included in the following results sections: Sample Size, Age, and Participants.

### Studies Characteristics

#### Sample Size

Among the 9 included studies, the smallest reported sample size was 10 [56] and the largest was 225 [58]. In quantitative studies, the number of participants ranged from 19 [51] to 122 [55]. In the mixed methods studies, the minimum number of participants was 12 [49] and the maximum was 225 [58]. Over the 9 studies, the average sample size was 73.1 (SD 72.4) individuals.

#### Age

Age is reported with varying degrees of accuracy depending on the methodologies used. The minimum age reported is 16 years [56], and the maximum is 63 years [49]. Based on the average ages reported by the studies [49,51-55,57,58], the total average age is 31.43 (SD 3.89) years.

#### Participants

The majority of studies (7/9, 78%) were based on samples of women diagnosed with endometriosis [51-56,58]. One study involved patients with a self-reported diagnosis [57], and one involved patients with a suspected diagnosis [49].

#### Countries

All the articles selected come from Western countries. Of these, the majority (5/10, 50%) were conducted in Europe, including 2 in France [52,53] and 3 in Germany [54-56]. Additionally, 4 studies took place in Australia [50,51,57,58]. Finally, 1 study has been carried out in Canada [49].

#### Types of DHIs

Of the 10 studies selected, the analysis revealed six distinct DHIs: (1) via a website [49], (2) health information recommendation features within web-based health applications [50], (3) an autonomous physical activity session using virtual reality video games for pain management [51], (4) a second intervention uses virtual reality and sensory stimuli for relaxation purposes to support patients in pain management (eg, Endocare) [52,53], (5) via a mobile application (ie, Endo-APP) [54-56], and (6) an SMS text messaging intervention (ie, EndoSMS) [57,58]. The first 3 DHIs were the subject of a single publication [49-51]. The DHI proposed by Abdulai et al [49] is a website that aims to provide information on dyspareunia and its management (eg, treatment) and to combat the stigma associated with sexual pain. The DHI of Li et al [50] aims to propose personalized disease management strategies (eg, therapy or solutions to manage pain, dietary information, etc) based on symptoms (ie, reported in an initial questionnaire) and an algorithm. The third DHI [51] aims to reduce pelvic pain through physical activity performed either autonomously through virtual reality or supervised by telehealth in comparison with a control group.

The other 3 have been the subject of several studies and publications [52-58]. Of these last 3 DHIs, the first concerns a virtual reality device (ie, Endocare) compared with a 2D version, which was tested in the laboratory [52] and then autonomously at home in a second study [53]. The second DHI is a mobile app called Endo-APP, which was used in 3 studies [54-56]. Two of these studies were conducted by the same research team, although they did not cite each other [54,55]. This can be explained by the fact that the manuscript for the first study [54] was submitted in 2022 but accepted in 2024. The third study [56], which used Endo-APP, is a qualitative study focusing on patient experience following the prescription of this app by a health care professional. Finally, the third DHI was based on sending SMS text messages (ie, EndoSMS) and includes 2 studies [57,58]. Sherman et al [58] evaluated the acceptance and feasibility of a 3-month SMS text messaging intervention designed in a previous study [57].

### Objectives, Interfaces, and Functionalities of the 6 DHIs Studied

The specific objectives pursued by these 6 DHIs vary from study to study. A total of 5 (83%) DHIs aimed to help manage pain or painful symptoms [49-56]. Among these 5 DHIs, 2 were based on a website but differ in their functionalities, with specific sections (eg, information on endometriosis, causes and treatments available for dyspareunia, and frequently asked questions) for Abdulai et al [49] and suggestions (eg, relaxation methods and dietary advice) based on initial responses to a questionnaire [50]. The other 2 DHIs used virtual reality but do not rely on the same functionalities. In the case of Lutfi et al [51], the interface and functionalities of the DHI depended on the participants’ choices (eg, relaxation application and video games). For Merlot et al [52,53], Endocare’s immersive intervention included auditory stimulation (ie, alpha and theta binaural beats and sounds inspired by nature) and visual stimulation by moving a laterally moving sphere. Finally, the Endo-APP mobile app was designed to help with this management. It features modules for monitoring symptoms, disease management (eg, educational content with articles and videos, exercise guides, and psychosocial support), or a personalized emergency plan for dealing with episodes of intense pain [54-56].

Overall, 4 (67%) DHIs aimed to provide information about endometriosis and strategies for managing the disease. They include the DHI proposed by Abdulai et al [49], that of Li et al [50], and the Endo-APP mobile app [54-56]. Another DHI targets this objective by being based on a unidirectional SMS text messaging broadcast, that is, with no possible response. The functionalities include the sending of 4 SMS text messages per week covering a variety of themes (eg, emotional health, social support, and information about the disease), and for some women, the possibility of adding a reminder to follow treatment. These authors also specify that this intervention can be personalized (ie, first name and time of receipt of messages) [57,58].

Furthermore, 2 (33%) interventions were intended to provide emotional and psychosocial support (ie, Endo-APP and EndoSMS). Finally, only the intervention proposed by Abdulai et al [49] sought to address the stigma associated with sexual pain. This information is summarized in Multimedia Appendix 2.

### Patient Feedback on DHI Design and Deployment

Of the 6 DHIs studied in this systematic review, 3 were developed with the support of women with endometriosis [49,50,57]. For the other 3 DHIs (ie, the virtual reality and telehealth interventions of Lutfi et al, Endocare, and Endo-APP), the articles do not mention patient involvement during development. However, 4 DHIs studied in 5 articles [49,50,56-58] were evaluated by women with endometriosis. These were the 3 DHIs developed with patients [49,50,57,58] and 1 DHI evaluated after it was approved for prescribing to patients [56]. Overall, 3 DHIs were evaluated with quantitative analysis [49,50,57,58] and 3 were analyzed with qualitative analysis [49,56-58]. More precisely, the DHI proposed by Abdulai et al [49] was evaluated both quantitatively and qualitatively, as was the DHI EndoSMS [57,58]. Endo-APP was evaluated qualitatively in the study by Zugaj et al [56] and the DHI by Li et al [50] quantitatively. The other DHIs (ie, Endocare and Lutfi et al DHI) do not seem to have been evaluated by the participants.

Quantitative evaluations allow objectifying patients’ user experience. A total of 3 DHIs were evaluated using this method. For example, the website proposed by Abdulai et al [49] is perceived as easy to use. Similarly, the study of Li et al [50] indicates that a quantitative evaluation of each proposed content was carried out. The details of each content are not reported. However, these authors noted a good user experience, although the number of returns was lower than expected. Similarly, Sherman et al [57] reported that the information provided in SMS text messages was useful and accessible to the majority of women with endometriosis, and so on. The same is true of the evaluation of this DHI under real-life conditions in terms of feasibility (eg, low attrition rate and few unsubscribes) and content acceptance [58].

Qualitative analysis identifies the limitations and potential for improvement of DHIs. A total of 3 DHIs were assessed using this methodology. For example, the study by Abdulai et al [49] enabled the research team to identify some minor problems (eg, comprehension of certain content such as graphics and icons), as well as features to be added (eg, search bar) or improved (eg, visibility of information on the home page). The study of Sherman et al [58] was positive overall, but some patients highlighted improvements (eg, disease information considered too general) and a greater need for personalization of the intervention (eg, content, frequency of SMS text messages, etc). Similarly, although positive, the study by Zugaj et al [56] highlights some limitations of the app, its content, or functionality (eg, time required for correct and regular use, concerns about security and data use, and content deemed redundant). They also pointed out missing functionalities (eg, possibility of exchanging information with peers) and made suggestions for improvement (eg, integration of information on diseases concomitant with endometriosis). Other DHIs have not been evaluated in terms of their development. However, the study by Merlot et al [53] showed that patients who received the intervention were significantly more satisfied than women in the control group.

### Efficiency to Manage Endometriosis

This systematic review highlights different types of benefits, such as physical, psychosocial, and self-management. A total of 5 studies [51-53,55,56] involving 3 different DHIs (ie, virtual reality and teleconsultation, Endocare, and Endo-APP) reported physical benefits. Regarding fatigue, the results are not homogeneous. Studies using the Endo-APP app [55] reported a significant reduction in fatigue compared to a control group. A DHI based on virtual reality (ie, Endocare) led to a reduction in fatigue, but there was no significant difference between the groups [53]. Finally, the other studies did not report any benefits in terms of fatigue. With regard to pain, these 5 studies reported a reduction in pain [51-53,55,56], regardless of the DHI used. It therefore seems that apps and virtual reality are relevant devices for managing physical symptoms, and specifically pain.

Regarding psychosocial benefits, 6 studies have reported benefits in this field. An improvement in quality of life was reported in 3 studies [53-55] based on 2 distinct DHIs (Endocare and Endo-APP). In the Endocare study (ie, virtual reality), there were no significant differences between the groups [53]. The other 2 studies reported significant improvements in Endo-APP users compared to the control group [54,55]. An improvement in self-efficacy in managing the disease and its symptoms was reported in 2 studies [55,56] involving a DHI (ie, Endo-APP). One study showed that patients found the website nonstigmatizing despite the subject matter [49]. Stress reduction was reported in 1 study [53] with no significant difference between groups, and 1 DHI has encouraged the adoption of active coping strategies [58]. Depressive symptoms were significantly reduced in 1 study [55], and an improvement in mood was reported in another [58]. A reduction in the nocebo effects associated with internet searches was observed in 1 study [56]. Better acceptance of the disease by certain women was reported in 1 study [56]. The same applies to the reduction of certain fears [56]. One study [58] reported a reduction in feelings of isolation and loneliness. The same applies to the increase in self-compassion [58].

In terms of the benefits of self-management, 3 studies report benefits, and 3 studies report on medication management. Overall, 3 types of DHIs (mobile app with personalized advice, EndoSMS, and Endo-APP) make it easier to access relevant information about the disease and change patients’ perceptions of it [50,56,58]. Studies about medication management all report beneficial effects, whatever the DHI (ie, Endo-APP, Endocare, and EndoSMS), related to the use of analgesics, for example [53,55], or to remembering to take treatment [58]. This information is summarized in Multimedia Appendix 2.

### Analysis of Selected Studies Based on the DEDHI Framework

#### Phase 1: Preparation

With the exception of Zugaj et al [56], all the studies mentioned at least 1 criterion corresponding to this first phase. However, there are major disparities between studies, with the criteria being met in 10%, with 1 in 10 criteria (eg, Rohloff et al [54]) to 80%, with 8 in 10 criteria (eg, Abdulai et al [49]) of cases. Among the criteria, a review of existing justificatory knowledge and feasibility were the most frequently reported in the studies. In contrast, criteria such as safety, privacy, and security were rarely reported.

#### Phase 2: Optimization

A total of 3 studies [50,51,54] reported criteria for the second phase. Li et al [50] and Rohloff et al [54] reported 54% (7/13) of the criteria, while the last study [51] reported 77% (10/13) of them. Criteria such as effectiveness, perceived benefits, and content quality were consistently reported. In contrast, perceived enjoyment was never mentioned, and criteria such as service quality, safety, privacy, and security were rarely taken into account. The other 7 studies [49,52,53,55-58] do not mention any elements relating to this second phase.

#### Phase 3: Evaluation

A total of 4 studies reported between 60% (6/10) and 80% (8/10) of the criteria for this third phase [50,52,53,55]. The other 6 studies [49,51,54,56-58] do not mention any of the criteria relating to this third phase of the DEDHI framework. As far as the evaluation criteria are concerned, effectiveness and perceived benefits are reported by all those meeting the criteria for this phase. Personalization was reported by only 1 of the 4 studies [50]. Moreover, privacy and security are rarely considered, as is the quality of service.

#### Phase 4: Implementation

A total of 2 articles met 77% (10/13) [50] and 62% (8/13) [56] of the criteria for the implementation phase of the DEDHI framework. The other 8 studies [49,51-55,57,58] did not mention any of the criteria for this phase. In these 2 studies, safety and service quality criteria are never mentioned. However, criteria such as personalization, perceived benefits, and content quality are the most regularly mentioned by studies responding to criteria from this fourth phase. The results of this section are summarized in Table 2.

**Table 2:** Analysis with Design and Evaluation of Digital Health Interventions (DEDHI) framework.

Phases, subphases, and subactivity				Abdulai et al [49]		Li et al [50]		Lutfi et al [51]		Merlot et al [52]		Merlot et al [53]		Rohloff et al [54]		Rohloff et al [55]		Sherman et al [57]		Sherman et al [58]		Zugaj et al [56]		
**Preparation phase (phase 1)**																								
	n (%)^a^			8 (80)		8 (80)		2 (20)		1 (10)		1 (10)		1 (10)		1 (10)		7 (70)		7 (70)		0 (0)		
	**Goals and tasks**																							
		Review existing justificatory knowledge	Y^b^ (p.^c^2)		Y (p.8)		Y (p.2)		Y (p.2)		Y (p.2)		Y (p.1158)		Y (p.1)		Y (p.2-3)		Y (p.2)		N^d^			
		Conceptual model	Y (p.2)		Y (p.8)		Y (p.4)		N		N		N		N		Y (p.7)		Y (p.2)		N			
		Feasibility and acceptability	Y (p.2)		Y (p.9)		N		N		N		N		N		Y (p.8)		Y (p.2)		N			
		Identify and optimization criterion	Y (p.5)		Y (p.9)		N		N		N		N		N		Y (p.8)		Y (p.7)		N			
	**Technical maturity**																							
		Research prototype that provides basic functionality	Y (p.4)		Y (p.9)		N		N		N		N		N		Y (p.8)		Y (p.2)		N			
	**Evaluation criteria**																							
		Ease of use	Y (p.7)		Y (p.9)		N		N		N		N		N		Y (p.9)		N		N			
		Adherence	Y (p.5)		N		N		N		N		N		N		N		Y (p.6)		N			
		Personalization	N		Y (p.8)		N		N		N		N		N		Y (p.8)		Y (p.2)		N			
		Safety	N		N		N		N		N		N		N		N		N		N			
		Privacy and security	Y (p.11)		Y (p.7)		N		N		N		N		N		N		N		N			
**Optimization phase (phase 2)**																								
	n (%)			0 (0)		7 (54)		10 (77)		0 (0)		0 (0)		7 (54)		0 (0)		0 (0)		0 (0)		0 (0)		
	**Goals and tasks**																							
		Conduct optimization trials	N		Y (p.9)		Y (p.2)		N		N		Y (p.1158)		N		N		N		N			
		Identify the best DHI^e^ configuration	N		N		Y (p.2)		N		N		N		N		N		N		N			
	**Technical maturity**																							
		Elaborated research prototype	N		Y (p.9)		Y (p.4)		N		N		Y (p.1158)		N		N		N		N			
	**Evaluation criteria**																							
		Effectiveness	N		Y (p.12)		Y (p.5)		N		N		Y (p.1161)		N		N		N		N			
		Perceived benefit	N		Y (p.9)		Y (p.5)		N		N		Y (p.1161)		N		N		N		N			
		Content quality	N		Y (p.9)		Y (p.4)		N		N		Y (p.1158)		N		N		N		N			
		Personalization	N		Y (p.9)		Y (p.4)		N		N		N		N		N		N		N			
		Perceived enjoyment	N		N		N		N		N		N		N		N		N		N			
		Aesthetics	N		N		Y (p.4)		N		N		N		N		N		N		N			
		Adherence	N		N		Y (p.4)		N		N		Y (p.1161)		N		N		N		N			
		Service quality	N		N		N		N		N		Y (p.1158)		N		N		N		N			
		Safety	N		N		Y (p.5)		N		N		N		N		N		N		N			
		Privacy and security	N		Y (p.7)		N		N		N		N		N		N		N		N			
**Evaluation phase (phase 3)**																								
	n (%)			0 (0)		6 (60)		0 (0)		8 (80)		7 (70)		0 (0)		6 (60)		0 (0)		0 (0)		0 (0)		
	**Goals and tasks**																							
		Confirm the effectiveness of an optimized DHI	N		N		N		Y (p.2)		Y (p.2)		N		Y (p.2)		N		N		N			
	**Technical maturity**																							
		Elaborated research prototype	N		Y (p.12)		N		Y (p.3)		Y (p.3)		N		Y (p.1)		N		N		N			
	**Evaluation criteria**																							
		Effectiveness	N		Y (p.12)		N		Y (p.8)		Y (p.7)		N		Y (p.5)		N		N		N			
		Perceived benefit	N		Y (p.12)		N		Y (p.8)		Y (p.7)		N		Y (p.5)		N		N		N			
		Adherence	N		N		N		Y (p.6)		Y (p.6)		N		Y (p.7)		N		N		N			
		Personalization	N		Y (p.12)		N		N		N		N		N		N		N		N			
		Service quality	N		N		N		Y (p.4)		N		N		N		N		N		N			
		Safety	N		N		N		Y (p.10)		Y (p.13)		N		N		N		N		N			
		Privacy and security	N		Y (p.7)		N		N		N		N		N		N		N		N			
		Accountability	N		Y (p.12)		N		Y (p.3)		Y (p.2)		N		Y (p.3)		N		N		N			
**Implementation phase (phase 4)**																								
	n (%)			0 (0)		10 (77)		0 (0)		0 (0)		0 (0)		0 (0)		0 (0)		0 (0)		0 (0)		8 (62)		
	**Goals and tasks**																							
		Develop a DHI product that can be implemented at a large scale in the health care market	N		Y (p.12)		N		N		N		N		N		N		N		N			
		Assess the long-term outreach and efficiency	N		Y (p.12)		N		N		N		N		N		N		N		Y (p.2254)			
		Update the DHI	N		Y (p.12)		N		N		N		N		N		N		N		Y (p.2260)			
	**Technical maturity**																							
		Elaborated research prototype	N		Y (p.12)		N		N		N		N		N		N		N		Y (p.2254)			
	**Evaluation criteria**																							
		Adherence	N		N		N		N		N		N		N		N		N		Y (p.2259)			
		Personalization	N		Y (p.12)		N		N		N		N		N		N		N		Y (p.2254)			
		Perceived benefit	N		Y (p.12)		N		N		N		N		N		N		N		Y (p.2258-2259)			
		Content quality	N		Y (p.9)		N		N		N		N		N		N		N		Y (p.2254)			
		Ethics	N		Y (p.5)		N		N		N		N		N		N		N		N			
		Service quality	N		N		N		N		N		N		N		N		N		N			
		Safety	N		N		N		N		N		N		N		N		N		N			
		Privacy and security	N		Y (p.7)		N		N		N		N		N		N		N		N			
		Accountability	N		Y (p.12)		N		N		N		N		N		N		N		Y (p.2254)			

^a^Percentage of completion for each phase, determined by the ratio between the number of criteria present and the total number of criteria expected for that phase.

^b^The criterion is identified, either explicitly stated or reasonably inferred from the content of the article.

^c^“p.” represents the page of the article where the criterion is mentioned.

^d^The criterion is not identified or cannot be reasonably inferred.

^e^DHI: digital health intervention.

## Discussion

### Overview

The aim of this systematic review was to characterize the design, quality, and efficiency of the digital technologies used to support women with endometriosis. This review highlights four important points: (1) the literature on this topic is at an early stage, although it is developing rapidly, as is the case for other chronic diseases; (2) digital interventions show physical, psychological, and self-management benefits, but the small number of studies and methodological variability limit the reliability of current evidence; (3) few studies mobilized a methodological framework to develop their intervention; and (4) analysis of the corpus using the DEDHI framework revealed that the majority of the studies essentially reported criteria relating to the first phase (development), leaving the later phases as yet unexplored.

Results of this systematic review highlight recent literature that is embedded in the growth of digital technology. Indeed, the least recent article selected in this corpus was published in 2022. In addition, the studies are mainly based in Western countries that have implemented policies to address this disease (eg, France [61], Canada [62], and Australia [63]), for instance, by supporting the funding of targeted research projects. This geographical focus may be linked to the fact that the disease has received increasing media coverage in recent years [64]. This geographical disparity may therefore contribute to the lack of international literature. This may result in an incomplete understanding of patient needs on a global scale. At the same time, the recent increase in DHIs and related publications follows the trend observed in endometriosis research, which has been steadily increasing since 2017 [65]. Finally, as reported in the results section, most of the studies conducted and the DHIs designed have been developed by the same research teams, which may result in some limitations of the literature on the subject. This literature is also the result of the evolution in the recommendations concerning the management of the negative effects of illness [4,5,66,67]. These recommendations were aimed, in particular, at improving the operational efficiency (eg, time management) of health care professionals [32,68] and the optimization of care and the quality of service provided to patients [69]. A growing trend is the use of digital health technology to enable patients with chronic illness to manage their disease over a long period of time [70], while developing their disease management skills [71]. These technologies are regularly used because of the freedom they offer (eg, use at home, at any time, or on any day) [72], both for the practitioner providing the care and for the patients benefiting from it. This systematic review illustrates precisely this point, since a number of applications and technologies (eg, websites, mobile apps, SMS text messaging, and virtual reality) are being used to optimize support for endometriosis. The use of digital technology for this disease is part of a wider movement concerning chronic pathologies, reflecting a paradigm shift in which patients become the central actors in their own care [73]. Several studies in this review indicate that patients were involved from the tool’s design stage [49,50,57,58] or during the evaluation phases of the tool designed to support them [49,50,56-58]. These studies show just how valuable it can be to involve patients from the earliest stages of device development—for example, to increase patient satisfaction [74]. The results of this study suggest that digital technologies can be useful in the treatment of endometriosis. However, they do not appear to be sufficient on their own to meet patients’ needs and could even have deleterious effects. Indeed, many women already report a sense of isolation and lack of recognition, both socially and within the health care system [10,75], which could be accentuated through digital technology. In this context, DHI could be seen as a complement to traditional care as part of a hybrid care model. This approach would exploit the advantages of digital tools while preserving the essential human dimension of care [76].

Studies in this systematic review reported physical, psychological, and self-management benefits of endometriosis. Indeed, some of the digital interventions in this corpus using digital technology [51-55] reported a reduction in pain, the main symptom of endometriosis [77]. Reduced pain can improve patients’ overall quality of life [78] and, therefore, has beneficial repercussions on other areas of patients’ lives, such as absenteeism from work [14] and social relationships [79]. Some studies in this corpus [54,55] reported a more global improvement in physical quality of life (eg, reduction in chronic pain) and psychological quality of life (eg, increased emotional well-being), while 1 study reported a reduction in feelings of loneliness and isolation [58]. Studies [53,55,58] have also shown a reduction in the use of analgesics. This could reduce treatment costs, which is particularly important in the context of endometriosis [80]. The DHIs presented in this systematic review seem to respond to the main challenges posed by endometriosis, whether related to the management of the disease or to its main consequences on physical, psychological, and social health, among others [81,82]. Furthermore, the articles reported an increase in patients’ feeling of being able to manage their symptoms [55,56]. Yet, self-efficacy is a key variable, having been shown in the literature to be an important determinant of the adoption and maintenance of health-enhancing behaviors (eg, health belief model) [83,84]. Indeed, a person with a high degree of self-efficacy will be more inclined to respond to a perceived threat [85] and to adopt relevant strategies to manage the symptoms of endometriosis. For example, the patient will be more likely to adopt health behaviors that improve her quality of life, such as physical activity [86] or a balanced diet [87]. To promote behavior change and management of this disease, future DHIs could draw on psychological and behavioral theories. Self-determination theory [88], the transtheoretical model of change [89], and the taxonomy of behavior change techniques developed by Michie et al [90] could offer valuable leads for the design of DHIs. Such models can help identify motivating factors, stages of change, and techniques adapted to users’ needs. Integrating these theoretical frameworks could improve the effectiveness and reproducibility of interventions. Finally, they provide a better understanding of the mechanisms of action underlying long-term user commitment and adherence. For instance, self-determination theory emphasizes autonomy, competence, and relationship as essential components of sustained motivation [91]. The transtheoretical model enables content to be adapted to the user’s stage of change. In this way, each individual could have a personalized intervention [92], as requested by some patients (eg, [58]). Using the taxonomy of behavior change techniques would enable a structured approach to the selection and presentation of behavioral strategies. 

Despite these advantages, a number of limitations need to be recognized. Most of the interventions examined were of short duration (up to 12 weeks [55]). It is therefore difficult to assess the durability of beneficial effects or the stability of user engagement over time. However, these elements appear to be crucial in understanding chronic diseases, such as endometriosis [93]. In addition, several studies [50,54-58] have proposed that intervention should be holistic, encompassing several dimensions of patient well-being (eg, dietary monitoring, stress management, and physical activity). While such an approach is recommended in the literature [94], this does not allow for the identification of the specific part played by the use of digital technology. Finally, as reported by Zugaj et al [56], the use of digital technologies does not seem to be suitable for all patients. This is why it seems necessary to pay particular attention to the personalization of devices [37,95] and take advantage of the variety of care modalities available in order to offer optimal support to each patient [94].

Among all the included studies and the 6 identified DHIs, 3 DHIs were developed with patient involvement [49,50,57]. Of these, only 2 articles [49,50] reported using a methodological framework to guide the development of a digital intervention and progressed to large-scale deployment. Nevertheless, 1 study [57] drew on various theories from social psychology (eg, theory of planned behavior [96]) to construct part of its intervention. After analyzing the studies using the DEDHI framework, it appears that almost all of them (9/10) focused on the criteria of phase 1 (preparation), leaving the subsequent phases unexplored. Furthermore, a detailed analysis of the phases revealed wide disparities in the way the criteria were addressed, particularly in this first phase. Thus, in the first phase, the criterion most often reported was the use of a literature review to justify technological choices and their positive effects on chronic pathologies. For example, Merlot et al [52] considered various meta-analyses on the use of virtual reality for chronic pain management in different pathologies, and Rohloff et al [54,55] did the same for the use of smartphones in pathologies with chronic pain. Nevertheless, even if the use of literature reviews is an essential step to support the development of intervention devices, it remains insufficient if criteria such as acceptability and acceptance of these devices are not taken into account because, as recommended by the DEDHI framework [37], they make it possible to adjust the intervention to the real expectations and needs of users. Moreover, considering “individual acceptability” would enable the adoption of a particular support strategy depending on the individual’s specific relationship with the technology [97]. On the other hand, the studies do not seem to have adapted their tools to the various standards and regulations in force concerning the use of digital technology in health (eg, General Data Protection Regulation). One explanation may lie in the fact that the research teams are more often than not made up of health care professionals and do not always include specialists in the development and implementation of these digital tools (eg, engineering research teams, lawyers, psychologists, and ergonomists). Yet, interdisciplinarity is generally considered to be one of the essential elements in medical innovation, particularly to meet the different needs of the market [98]. The ever-increasing call for research into health care innovations [99] may lead research teams to neglect certain essential stages of development (eg, optimization, evaluation, and implementation), which can compromise the ownership and sustainability of the intervention device and associated benefits [37]. Li et al [50], who highlight the advantages of mobilizing a clearly defined methodological framework to think through the implementation of a technology with potential users, support this view. Yet, some of the included studies collected patient perceptions during the development or trial phases of DHIs. For example, 3 studies [49,56,58] have highlighted that patient satisfaction and engagement were linked to perceptions of ease of navigation, clarity of content, and perceived credibility of information. Conversely, problems such as excessive text content [49], poor section visibility [50], or a lack of personalization [58] were seen as potential barriers to engagement with the intervention or continued use. These results, therefore, underline the need to systematically involve the various stakeholders in the development process. In addition, they also highlight the importance of user testing in order to limit identified obstacles and support potential long-term use.

### Strengths and Limitations of This Review

This systematic review has 4 main strengths. First, the PRISMA methodology and its stages were applied [41]. Second, the quality of each study was assessed with an adjusted evaluation of the risk of bias according to the experimental design used [100]. Third, each article was evaluated by several researchers, a discussion was organized to harmonize assessments, and an intraclass correlation coefficient calculation [101] was performed for the risk of bias. Fourth, several databases were mobilized to improve the comprehensiveness of the search [100].

Several limitations can also be identified. First, a meta-analysis could not be carried out due to the heterogeneity of experimental designs implemented, the measurements performed, and the results observed in the review analysis [102]. This methodological variability has limited the possibility of producing a quantitative synthesis of results or drawing definitive conclusions about the effectiveness of specific digital interventions. Thus, future systematic reviews or meta-analyses on the effects of digital interventions will need to be conducted, particularly on variables of interest in the context of endometriosis (eg, perceived pain, quality of life, and anxiety). Second, the published literature often reports positive and significant results [103], and there is not always a control group to put the results into perspective. This bias may have influenced the overall impression of the effectiveness or feasibility of the DHIs presented in this review. As a result, these findings may provide an overestimated picture of the actual benefits or readiness of these tools for wider implementation, while underrepresenting the difficulties or limitations encountered in practice. Third, the growing interest in digital and associated technologies is such that the search equation used may not have identified some articles mobilizing terminologies different from ours [104]. Fourth, following the selection process, 10 studies were retained out of the 381 articles identified. While this highlights the current state of research, it also limits the scope of the conclusions of this systematic review and the generalizability of the results. However, the recent increase in the number of studies on the subject calls for clarification of the methodological framework used to develop interventions and the associated digital tools, which should help to improve the reproducibility of interventions. Finally, the studies in this review were mainly conducted in Western countries, which raises questions about the transferability of the results, particularly given the difficulties of accessing and using DHIs linked to cultural, economic, and physical factors [105]. It is possible that in some parts of the world, access to this type of technology and the internet is more limited, which could reduce the reach of these digital solutions. For example, without the support of health authorities, the financial cost of accessing these digital resources may be too high and restrict access. Unequal access to digital resources may also hinder the development of digital literacy (ie, digital skills and the skills to understand and use content available on the internet [106]). These elements are identified as potential barriers to the use of DHIs [107]. It therefore seems necessary for international research teams to focus on the development of digital health devices. The approach could be to develop or adapt existing frameworks to the cultural and contextual specificities of their implementation to ensure greater accessibility and usability for diverse groups.

### Research Implications

In light of these challenges, future research could potentially focus on several key priorities. First, longitudinal studies appear necessary to better understand the mechanisms underlying the sustained use of digital health tools [94]. Second, more rigorous and interdisciplinary research (eg, randomized controlled trials) with larger samples and standardized outcome measures would also appear to be relevant. Carried out in this way, they would ensure reproducibility while strengthening the evidence base for the effectiveness of digital interventions. Third, qualitative studies exploring patients’ experiences, expectations, and perceptions of digital health technologies could be carried out. This could complement the results of quantitative evaluations of content or user experience [108]. Fourth, cross-cultural studies could be conducted to assess the transferability of these interventions for different patient profiles (eg, age, sociodemographics, and digital literacy), as well as for different cultural, geographical, and socioeconomic contexts [109]. Fifth, future studies could explore hybrid models of care to better meet patients’ needs and evaluate the effectiveness of this type of support. Sixth, future studies could be based on the study of behavior change theories or on the identification of behavior change techniques used by DHI. Finally, future studies should systematically apply methodological frameworks, such as DEDHI, to frame the development and implementation process. All relevant phases and criteria would then be transparently reported in their publications.

### Conclusion

This systematic review highlights the current interest in using digital technology to support patients with endometriosis. It appears promising and offers advantages in terms of patient care. However, the development of digital devices is rarely based on a methodological framework that would direct each stage of development to maximize patient involvement and, ultimately, improve their overall quality of life.

Overall, this systematic review has highlighted a number of elements for the development of digital devices for the management of endometriosis, from the initial design phases through to market implementation and appropriation by patients. Three points seem crucial to develop interventions mobilizing digital technology and encourage patients with endometriosis to appropriate these tools: (1) mobilizing a methodological framework tailored to the context and specific to the development of support with digital devices, (2) relying on a multidisciplinary team (ie, doctors, engineers, ergonomic psychologists, and lawyers) capable of addressing every aspect of the development of the digital device, and (3) involving all stakeholders in the management of endometriosis (ie, patients, algologists, gynecologists, psychologists, and physiotherapists) in the design. These few points could serve as a guideline for outlining the contours of a global research reflection on the development of interventions using digital tools in the context of endometriosis, aimed at improving the quality of patient care. Developed using this approach, these digital tools place patients at the heart of the interventions by offering them not only concrete tools to better manage their health but also a space to share their experiences with other patients concerned. These elements help to foster health empowerment and, ultimately, pain management [110]. These digital tools can help to change the way patients are supported, moving away from “traditional medical care” to “holistic care,” which takes into account all the dimensions of a patient’s health—physical, mental, and social.

## Appendix

Multimedia Appendix 1 PRISMA checklist.

Multimedia Appendix 2 Details of studies included and results of interventions (n=10).

## Abbreviations

DEDHI: Design and Evaluation of Digital Health Interventions
DHI: digital health intervention
PRISMA: Preferred Reporting Items for Systematic reviews and Meta-Analyses
MMAT: Mixed Methods Assessment Tool
