# Supplementary material for: Endometriosis Support and Development of Digital Technology–Based Interventions: Systematic Review
Source: JMIR Hum Factors. 2025 Oct 14;12:e71859. doi: 10.2196/71859 (PMC12569497; doi:10.2196/71859)
Supplement: Multimedia Appendix 2 [file humanfactors_v12i1e71859_app2.docx]

**Table S1.** Details of studies included and results of interventions (n=10).

| **Authors** | **Country** | **Methods** | **Diagnosis**  **Effectif (n)** | **Digital technologies** | **Risk of bias*** | **Objective of DHI** | **Co-design** | **Fonctionnalities** | **Efficiency** |
| --- | --- | --- | --- | --- | --- | --- | --- | --- | --- |
| Isolated initiative of an intervention test using a digital tool | | | | | | | | | |
| Abdulai et al. [49] | Canada | Mixed-  methods | Patients diagnosed with or clinically suspected of having endometriosis    N=12 | Website | 4.5  (90%) | Information on dyspareunia and its management  Reducing the stigma attached to sexual pain | Multidisciplinary team: scientists, healthcare professionals and patients | Information on endometriosis, painful sexual relations, causes of pain, treatments available, additional resources, frequently asked questions | No stigmatisation |
| Li et al. [50] | Australia | Case study | N/A | development of the information recommenda- tion  functionalities on the website | 2 (40%) | Personalised disease self-management strategies based on symptoms and an algorithm | Multi-disciplinary team: patients, members of associations, researchers and health informatics specialists | Targeted suggestions based on responses to a questionnaire | Better identification of relevant strategies for managing the disease |
| Lutfi et al. [51] | Australia | Quantitative  Pilot RCTs | Endometriosis diagnosis  N = 19  (telehealth, n = 7; VR, n = 8; control, n = 4) | Telehealth (1h) vs VR (1h) vs Control  (continue with their activities of daily living) | 3.5 (70%) | Reducing pelvic pain by physical activity performed autonomously in VR or supervised by telehealth | N/A | Depends on the patient's choice of game in VR | Pain benefits |
| Testing an intervention using virtual reality (Endocare) | | | | | | | | | |
| Merlot et al. [52] | France | Quantitative  RCTs | MRI endometriosis Diagnosis  N= 45 Endocare = 23, Control = 22 | VR vs Control (same situation but in 2D on tablet) | 3.5 (70%) | Reduce chronic pelvic pain | N/A | Auditory and visual stimulation | Pain benefits |
| Merlot et al. [53] | France | Quantitative  RCTs | Endometriosis diagnosis  N=102  Endocare = 51  Control = 51 | VR vs Control (same situation but in 2D ) | 3.5 (70%) | Reduce chronic pelvic pain | N/A | Auditory and visual stimulation | Benefits pain + fatigue + reduced stress + reduced use of painkillers |
| Testing an intervention using a mobile app (Endo-App) | | | | | | | | | |
| Rohloff et al. [54] | Germany | Quantitative | Prior diagnosis of endometriosis  N=106  Endo-APP = 64  no user = 42 | Application | 3.5 (70%) | Improving patients' quality of life with content that is complementary to standard care | N/A | monitoring diary, exercise guide, nutritional advice, educational content, psychosocial support, stress management and coping techniques, personalised emergency plan for pain management, etc. | Improved quality of life |
| Rohloff et al. [55] | Germany | Quantitative  Pilot RCTs | Medical diagnosis of endometriosis  N=122 | Application + standard care VS usual care | 4.5( 90%) | Improving patients' quality of life with content that is complementary to standard care | N/A | monitoring diary, exercise guide, nutritional advice, educational content, psychosocial support, stress management and coping techniques, personalised emergency plan for pain management, etc. | Reduction in pain + fatigue + symptoms + improvement in pain self-efficacy + reduction in depressive symptoms and increase in quality of life + reduction in the use of analgesics |
| Zugaj et al. [56] | Germany | Qualitative | Patients diagnosed with endometriosis and who have received a prescription for the DiGa application: ‘Endo-App©’  N=10 | Application | 5 (100%) | Improving patients' quality of life with content that is complementary to standard care | N/A | monitoring diary, exercise guide, nutritional advice, educational content, psychosocial support, stress management and coping techniques, personalised emergency plan for pain management, etc. | Improved perception of pain + reduced fears and increased self-efficacy + change in perception of illness |
| Testing an intervention using SMS (EndoSMS) | | | | | | |  |  |  |
| Sherman et al. [57] | Australia | Mixed-  methods | Diagnosed with endometriosis (self-reported)  N=17 | SMS | 4 (80%) | Providing SMS to help manage the disease, emotional management and social support | Patients and healthcare professionals | 4 text messages a week on different themes: emotional health, empowerment, interpersonal issues, general information, physical health and reminders about medication compliance. Personalized with first name and reception time. |  |
| Sherman et al. [58] | Australia | Mixed methods integrated into RCTs | Clinically diagnosed with endometriosis  N=225  SMS = 110  Waitlist = 115 | SMS | 4.5 (90%) | Providing SMS to help manage the disease, emotional management and social support | Patients and healthcare professionals | 4 text messages a week on different themes: emotional health, empowerment, interpersonal issues, general information, physical health and reminders about medication compliance. Personalized with first name and reception time. | Reducing social isolation and loneliness+ compassion for self + adoption of active strategies + improvement mood + useful information for managing the disease and greater autonomy + reminders to take medication |

Notes. *APP: application; DiGa: Digital health applications; DTx: Digital therapeutics; N/A: Not Available; MRI: Magnetic Resonance Imaging; SMS: Short Message Service; RCTs: randomized control trials study; VR: virtual reality.*

**A higher percentage indicates a lower risk of bias*.
